# Assessing the between-country genetic correlation in maize yield using German and Polish official variety trials

**DOI:** 10.1007/s00122-022-04164-2

**Published:** 2022-07-13

**Authors:** Waqas Ahmed Malik, Harimurti Buntaran, Marcin Przystalski, Tomasz Lenartowicz, Hans-Peter Piepho

**Affiliations:** 1 Biostatistics Unit, Institute of Crop Science, University of Hohenheim, Fruwirthstrasse 23, 70599 Stuttgart, Germany; 2 Research Centre for Cultivar Testing, Słupia Wielka 34, 63-022 Słupia Wielka, Poland

## Abstract

**Key message:**

We assess the genetic gain and genetic correlation in maize yield using German and Polish official variety trials. The random coefficient models were fitted to assess the genetic correlation.

**Abstract:**

Official variety testing is performed in many countries by statutory agencies in order to identify the best candidates and make decisions on the addition to the national list. Neighbouring countries can have similarities in agroecological conditions, so it is worthwhile to consider a joint analysis of data from national list trials to assess the similarity in performance of those varieties tested in both countries. Here, maize yield data from official German and Poland variety trials for cultivation and use (VCU) were analysed for the period from 1987 to 2017. Several statistical models that incorporate environmental covariates were fitted. The best fitting model was used to compute estimates of genotype main effects for each country. It is demonstrated that a model with random genotype-by-country effects can be used to borrow strength across countries. The genetic correlation between cultivars from the two countries equalled 0.89. The analysis based on agroecological zones showed high correlation between zones in the two countries. The results also showed that 22 agroecological zones in Germany can be merged into five zones, whereas the six zones in Poland had very high correlation and can be considered as a single zone for maize. The 43 common varieties which were tested in both countries performed equally in both countries. The mean performances of these common varieties in both countries were highly correlated.

**Supplementary Information:**

The online version contains supplementary material available at 10.1007/s00122-022-04164-2.

## Introduction

Variety examination offices evaluate the performance of newly bred varieties for their value of cultivation and use (VCU) before their addition to the national list and admission for commercial use in a country. The main objective of testing is to assess the relative phenotypic performance of the new varieties and only release the best varieties for commercial use. For example, every year on average 20 new maize varieties are registered in Germany. Before registration, they are tested for 2 to 3 years at up to 25 locations in the main growing area of maize in Germany. Similarly, in Poland, on average 30 new maize varieties are registered, after testing in official trials at up to 32 locations over 2 to 3 years. According to Laidig et al. (2008), 50% of the candidate varieties are usually withdrawn by the breeder after the first testing year, and only 25–30% reach the last year and are able to get registration.

Maize is grown across a wide range of environments, and the growth of plants and the harvested product depend on the climatic and environmental conditions. An important objective of plant breeders is to develop broadly adapted varieties for a wider target region. On the basis of similarity in the agroclimatic conditions, a larger region can be subdivided into zones. These zones can extend beyond the political or national boundaries and can be regarded as mega-environments in which genotypes perform relatively homogeneously (Gauch and Zobel 1997). Kleinknecht et al. (2013) and Piepho and Möhring (2005) presented an approach showing how best linear unbiased prediction (BLUP) can be used for selection of genotypes for zones or mega-environments. The BLUP method allows the borrowing of information across zones or mega-environments to exploit genetic correlation between zones (Buntaran et al. 2019, 2020), thereby providing more accurate estimates of genotype performance as compared to best linear unbiased estimation (BLUE), which cannot exploit such correlation.

The predictive ability of models can be improved by incorporating soil or environmental covariates (van Eeuwijk et al. 2016). Usually, in the analysis of multi-environment trial (MET) data, the variety-specific regression terms for covariates are taken as fixed effects (Denis 1988). However, when the target region is divided into zones and variety effects are modelled as random to borrow strength across zones, then variety-specific regression coefficients must be modelled as random, giving rise to random coefficient models (Longford 1993; Buntaran et al. 2021).

This paper aims to assess the genetic correlation between the maize agroecological zones of Germany and Poland using official variety trials of maize from 1987 to 2017. The 43 common varieties allow the assessment of the genetic correlation between the two countries and among agroecological zones in these countries. Environmental covariates are also incorporated into the statistical models to achieve better fit and predictions. The rest of the paper is structured as follows. First, we describe the datasets from German and Poland official registration trials for maize. The models are then outlined in detail, followed by an illustration of results for maize trials from Germany and Poland. Finally, a discussion on the results is presented, focusing on the correlation between agroecological zones of Germany and Poland.

## Materials and methods

### Datasets

**Fig. 1:**
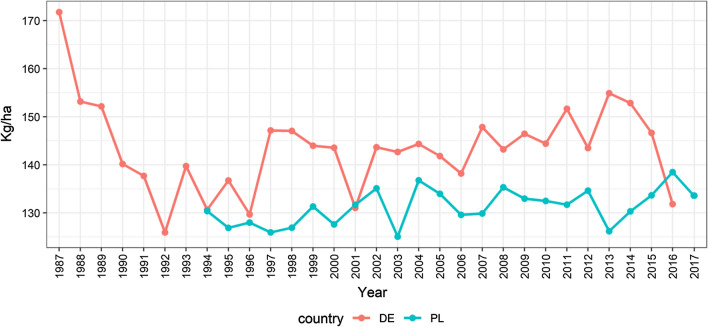
Application of nitrogen in field trials in Germany and Poland from 1987 to 2018

In this study, datasets from the official grain maize variety trials of the Bundessortenamt (Hannover) in Germany from 1987 to 2016 and the Research Centre for Cultivar Testing (COBORU) in Poland from 1994 to 2017 were used. The German trials were conducted for assessing the value for cultivation and use (VCU), whereas the Polish trials were both VCU and post-registration trials (PDO). The trait yield in dt/ha is considered for the analysis. All trials from Germany were laid out as randomised complete block designs with three replications, while trials from Poland were laid out in a 1-resolvable design with three replicates. The crop management in the trials included standard fertilisation adapted to conditions in each location. The applied levels of nitrogen fertilisation are given in Fig. 1.

**Fig. 2:**
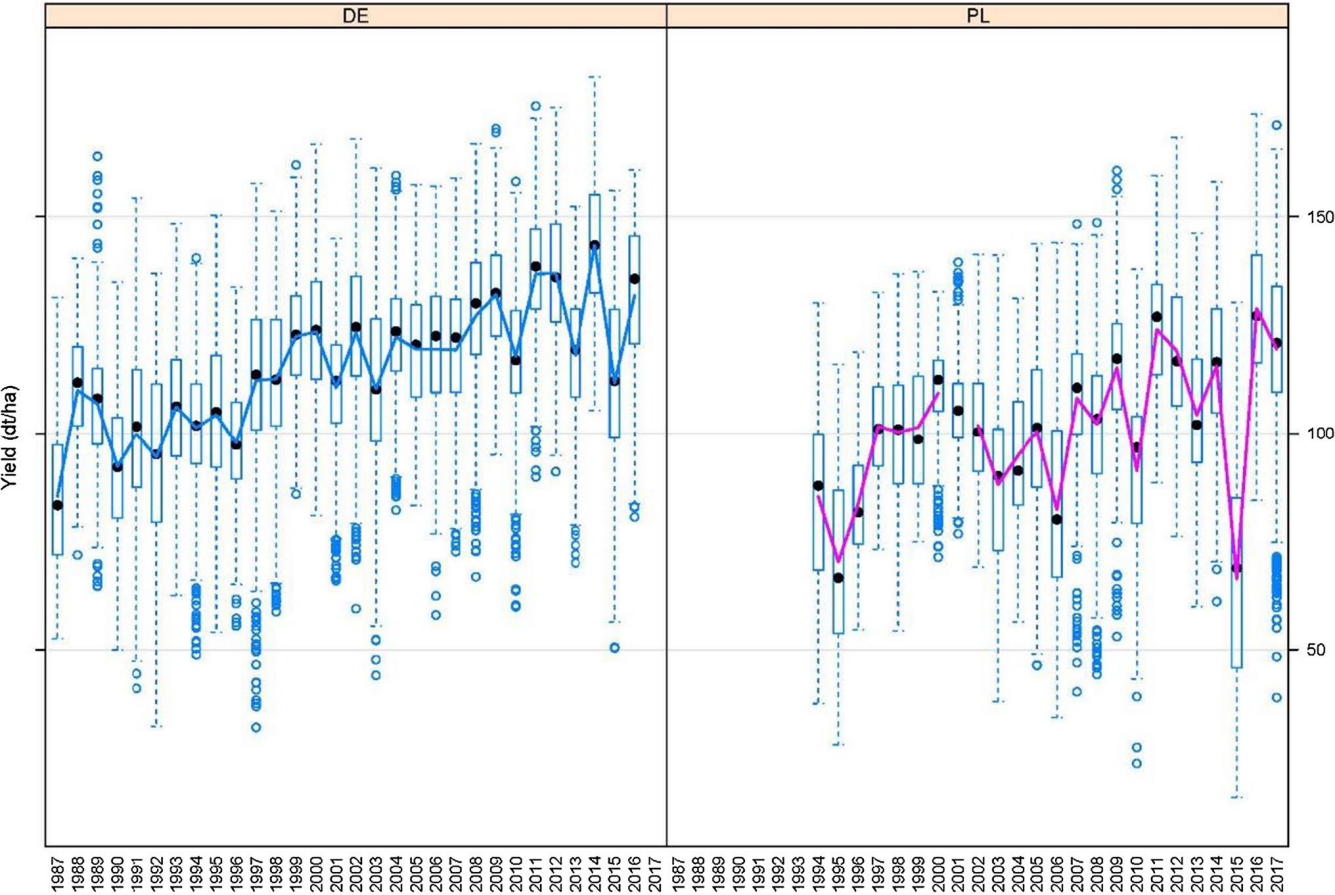
Year-wise yield (dt/ha) of maize field trials in Germany (left) and Poland (right) from 1987 to 2017. The lines represent yearly mean yield

The datasets were non-orthogonal because new varieties were tested for two or three years, and after each year, some varieties were withdrawn, whereas new varieties entered the trials. In any particular year, the same set of varieties was tested at each location in that year. Therefore, data within years are balanced for varieties and location; however, data are unbalanced for varieties by years. Some basic information about the datasets is given in Table 1, whereas the year-wise yields for both countries are plotted in Fig. 2. The lines in Fig. 2 represent year mean yield for each country. An increasing trend can be seen in both countries. Between 1987 and 2016, there were 43 varieties tested in both countries. A list of common varieties along with testing year is given in Table 2.

**Table 1:** Basic information on the yield trial data of grain maize from Germany and Poland

Country	Years	Observations	Varieties	No. of locations	Environments (Year × Loc.)	Zones
Germany						
VCU	1987 − 2016	15,642	350	121	770	22
Poland		15,087	634	32	408	6
VCU & PDO	1994 − 2000	3012	123	23		
VCU	2000 − 2017	7542	460	19		
PDO	2000 − 2017	4533	139	26

**Table 2:** List of 43 common varieties that were tested in Germany and Poland from 1987 to 2017

Variety	Germany	Poland
AMADEO	2002,2003, 2004, 2005, 2006, 2007, 2008	2005, 2006, 2007, 2008
AMARYL	2006, 2007	2010
AMOROSO	2003, 2004	2014
BENEDICTIO KWS	2014, 2015	2017
CALAS	1998, 1999	2003
CARLTON	1992, 1993	1994, 1995, 1996
DELITOP	2001, 2002, 2003, 2004, 2005, 2006,	2005, 2006, 2007
DKC 2960	2004, 2005, 2007, 2008	2008, 2009, 2010
ES ALBATROS	2010, 2011	2012, 2013
ES ANAMUR	2002, 2003	2006
ES METRONOM	2012, 2013, 2015, 2016	2015
ES PAROLI	2003, 2004, 2006, 2007	2006, 2007, 2008, 2009, 2010, 2011
EUROSTAR	1998, 1999	2001, 2002, 2003, 2004, 2005
FIGARO	1987, 1988, 2014, 2015, 2016	2017
FJORD	1997, 1998, 1999, 2000, 2001, 2002	2001, 2002
GRANEROS	2000, 2001	2006, 2007
HEXXER	1998, 1999	2006, 2007
KAMPALA	1994, 1995, 1996	2003
KORNELI	2001, 2002	2007, 2008
LG 3258	2007, 2008, 2009, 2010, 2011, 2012	2012, 2013, 2014
LG 3226	1999, 2000, 2002, 2003, 2004, 2005, 2006	2003, 2004, 2005
LUIGI CS	2008, 2009, 2011, 2012, 2013, 2014	2012, 2013
MONCADA	2002, 2003	2004
MONUMENTAL	1998, 1999	2003, 2004, 2005, 2006, 2007, 2008
NATACHA	1989, 1990, 1991	1994
NK NEKTA	2005, 2006, 2009, 2010	2008, 2009, 2010, 2011, 2012, 2013, 2014
P 8000	2007, 2008	2011
P 8329	2014, 2015	2017
P 8400	2009, 2010, 2011, 2012, 2013, 2014, 2015	2013
PR39G12	1999, 2000	2003
PR39H32	2000, 2001	2003, 2004, 2005, 2006, 2007, 2008
RICARDINIO	2009, 2010	2010, 2011, 2012, 2013, 2014, 2015, 2016
RIVALDINIO KWS	2011, 2012	2014, 2015, 2016, 2017
RIVALDO	1998, 1999, 2001, 2002, 2003, 2004	2015, 2017
ROMARIO	1997	2003, 2004
SANTIAGO	1998	1998, 1999
SILAS	2007, 2008	2008
SUSETTA	2014, 2015	2017
SY TELIAS	2014, 2015	2017
TIBERIO	2004, 2005	2010, 2011, 2012
TONINIO	2010, 2011	2014, 2015
VERITIS	1999, 2000	2003, 2004, 2005
ZIDANE	2005, 2006, 2008, 2009, 2010, 2011	2009, 2010

**Fig. 3:**
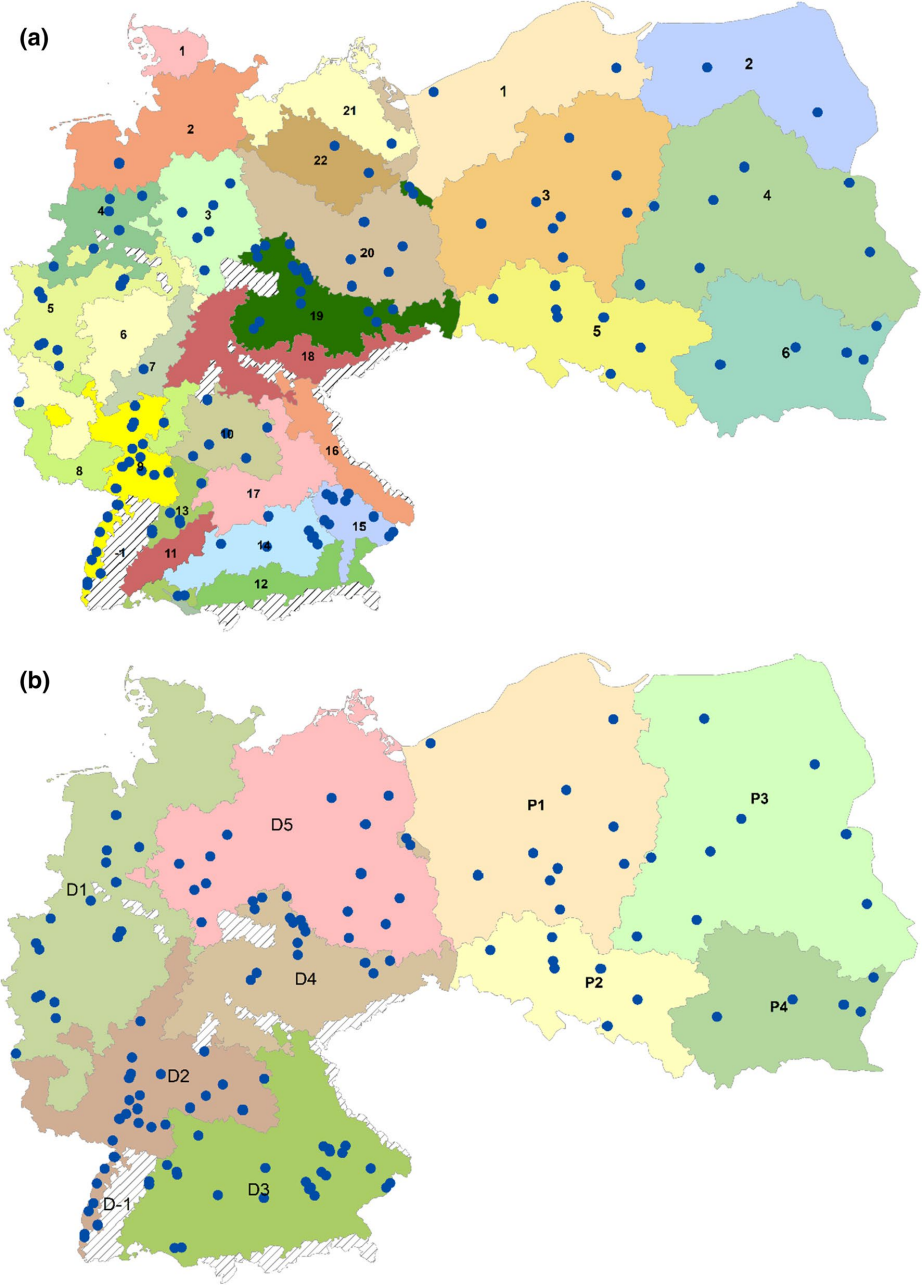
Maize agroecological zones of Germany and Poland. **a** Maize-growing area in Germany is classified into 22 agroecological zones, while maize-growing area in Poland is classified into 6 zones. **b** merged agroecological zones

Based on the agroclimatic condition and soil type, the maize-growing area in Germany has been classified into 22 agroecological zones (Graf et al. 2009; Maiskomitee 2022). Similarly, the maize-growing area in Poland is classified into six zones (Fig. 3). Each location used for official maize trials was assigned to one of these zones. Zones with fewer locations were merged with the neighbouring zone because some zones were represented by only a few locations, as depicted in Fig. 3. Also, the merging of the zones was performed after analysing the data and looking into the genetic correlations between zones (results not shown). For example, Zone 1 and Zone 3 in Poland have a higher correlation as compared to the correlation between Zone 1 and Zone 2. Therefore, Zone 1 and Zone 3 were merged to form one zone. This zone merging resulted in five zones in Germany and four zones in Poland, which were subsequently used in the analysis.

## Models for the analysis of data from Germany and Poland maize variety trials

The basic model for analysis of multi-environment trial data is expressed as:

1 $${yield}_{ijk}=\mu +{g}_{i}+{s}_{j}+{y}_{k}+{(sy)}_{jk}+{(gs)}_{ij}+{(gy)}_{ik}+ {(gsy)}_{ijk}^{^{\prime}}$$

where ${yield}_{ijk}$ is the mean yield of the *i*th genotype (or variety) in the *j*th location (or site) and the *k*th year $(i = 1, \ldots ,n_{g} ;\;j = 1, \ldots ,n_{s} ;\;k = 1, \ldots ,n_{y} )$, μ is the overall mean, ${g}_{i}$ is the main effect of the *i*th genotype, ${s}_{j}$ is the main effect of the *j*th location, ${y}_{k}$ is the main effect of the *k*th year, ${(sy)}_{jk}$ is the interaction effect of the *j*th location and *k*th year, ${(gs)}_{ij}$ is the interaction effect of the *i*th genotype and *j*th location, ${(gy)}_{ik}$ is the interaction effect of the *i*th genotype and *k*th year, and ${(gsy)}_{ijk}^{^{\prime}}$ is the sum of the interaction effect of the *i*th genotype, *j*th location and *k*th year, and the residual for the mean yield ${yield}_{ijk}$.

### Country and agroecological zone levels analyses

Since locations within countries are taken to be a representative sample within countries, Model 1 can be extended as follows, including a country main effect and its interaction with other main effects.

2 $${yield}_{ijlk}=\mu +{c}_{l}+{g}_{i}+{(gc)}_{il}+{y}_{k}+{(yc)}_{kl}+{(gy)}_{ik}+{(gyc)}_{ikl}+{s}_{jl}+{(sy)}_{jlk}+{(gs)}_{ijl}+{(gsy)}_{ijlk}^{^{\prime}}$$

where ${c}_{l}$ is the effect of the *l*th country $(l=1,\dots ,{n}_{l})$, ${s}_{jl}$ is the effect of the *j*th location nested within the *l*th country, ${(gc)}_{il}$ is the interaction effect of the *i*th genotype and the *l*th country, $({yc)}_{kl}$ is the interaction effect of the *k*th year and the *l*th country, ${(gyc)}_{ikl}$ is the interaction effect of the *i*th genotype, *k*th year and the *l*th country, and ${(gsy)}_{ijlk}^{^{\prime}}$ is the sum of the interaction effect of the *i*th genotype, *j*th location within the *l*th country and the *k*th year, and the residual.

In general, to get the genotype-by-country or genotype-by-zone means, we can model the main effects for genotype and country, and their interaction as fixed effects, and the rest of the effects as random, i.e. location, year and any interaction effects with year and location. Stacking effects of the same type into vectors (e.g. all location main effects, etc.), we may assume that these are independently normally distributed with zero mean and variance–covariance structures, ${\mathbf{G}}_{s}$, ${\mathbf{G}}_{y}$, ${\mathbf{G}}_{sy}$, ${\mathbf{G}}_{yc}$, ${\mathbf{G}}_{gs}$, ${\mathbf{G}}_{gy}$, ${\mathbf{G}}_{gyc}$ and ${\mathbf{R}}_{gsy}$, respectively.

In the case of exploiting the genetic correlation between countries, the vector *g* of genotype main effect is modelled as random with the assumption $g\sim N(0,{\mathbf{G}}_{g})$. In this case, the variance of the main effect is equivalent to the genetic covariance between the two countries. For the country level analysis, the fixed genotype effect (FG) model and the random genotype effect (RG) model were fitted.

The different agroecological zones for maize in Germany and Poland can be used for the agroecological zone-level analysis. In this case, the country effect $({c}_{l})$ in Model 2 is replaced by the zone effect $({z}_{l})$, and all other effects stay the same. Thus, Model 2 becomes

3 $${yield}_{ijlk}=\mu +{z}_{l}+{g}_{i}+{(gz)}_{il}+{y}_{k}+{(yz)}_{kl}+{(gy)}_{ik}+{(gyz)}_{ikl}+{s}_{jl}+{(sy)}_{jlk}+{(gs)}_{ijl} +{(gsy)}_{ijlk}^{^{\prime}}$$

where ${z}_{l}$ is the effect of the *l*th zone. As some agroecological zones were represented by only a few locations, we grouped a few of the neighbouring zones into mega-environments, which resulted in five zones in Germany and four zones in Poland (see Fig. 3). Another extension of Model 3 can be made by considering location nested within zones and zones nested in countries. However, we are not considering this model because our objective here is to determine the correlation between the zones in the countries without considering the effect of the country. For zone-level analyses, only the RG model was fitted since the aim is to exploit the genetic correlation between agroecological zones. Furthermore, in the RG model, different variance–covariance structures for genotype and genotype × country $(gc)$ or genotype × zone $(gz)$ effects were fitted. The models with fixed genotype and random genotype effects are given in Table 3, while Table 4 contains an overview of variance–covariance structures for each term in the models.

**Table 3:** Two fixed genotype effect (FG) and six random genotype effect models

Model	Fixed part	Random part
FG	$\mu +{c}_{l}+{g}_{i}+{(gc)}_{il}$	${y}_{k}+{(yc)}_{kl}+{(gy)}_{ik}+ {(gyc)}_{ikl}+ {s}_{jl}+{(sy)}_{jlk}+{(gs)}_{ijl}$
FGC	$$\begin{aligned} \mu + & c_{l} + g_{i} + (gc)_{{il}} + \beta _{1} x_{{1jl}} + \eta _{l} x_{{1jl}} \\ + & \beta _{2} x_{{2i}} + \beta _{3} x_{{3i}} \\ \end{aligned}$$	Same random part as in FG
RG	$\mu +{c}_{l}$	${g}_{i}+{(gc)}_{il}+{y}_{k}+{(yc)}_{kl}+{(gy)}_{ik}+ {(gyc)}_{ikl}+ {s}_{jl}+{(sy)}_{jlk}+{(gs)}_{ijl}$
RGC	$\mu +{c}_{l}+ {\beta }_{1}{x}_{1jl}+{\eta }_{l}{x}_{1jl}+{\beta }_{2}{x}_{2i}+{\beta }_{3}{x}_{3i}$	Same random part as in RG
RC1	Same fixed part as in RGC	$({a}_{i}+{b}_{i}{x}_{1jl})+({p}_{il}+{q}_{il}{x}_{1jl})+{y}_{k}+{(yc)}_{kl}+{(gy)}_{ik}+ {(gyc)}_{ikl}+ {s}_{jl}+{(sy)}_{jlk}+{(gs)}_{ijl}$
RC2	Same fixed part as in RGC	${g}_{i}+({p}_{il}+{q}_{il}{x}_{1jl})+{y}_{k}+{(yc)}_{kl}+{(gy)}_{ik}+ {(gyc)}_{ikl}+ {s}_{jl}+{(sy)}_{jlk}+{(gs)}_{ijl}$
RC3	Same fixed part as in RGC	$({a}_{i}+{b}_{i}{x}_{1jl})+{(gc)}_{il}+{y}_{k}+{(yc)}_{kl}+{(gy)}_{ik}+ {(gyc)}_{ikl}+ {s}_{jl}+{(sy)}_{jlk}+{(gs)}_{ijl}$
RC4	Same fixed part as in RGC	$({p}_{il}+{q}_{il}{x}_{1jl})+{y}_{k}+{(yc)}_{kl}+{(gy)}_{ik}+ {(gyc)}_{ikl}+ {s}_{jl}+{(sy)}_{jlk}+{(gs)}_{ijl}$

The index *i* refers to the *i*th genotype, *j* refers to the *j*th location, and *l* refers to the *l*th country or zone. The country term, $c$, is replaced with $z$ for the agroecological zone level analysis

**Table 4:** Variance–covariance structures for each random term in the models

Random term	Variance–covariance structure	Remarks
$g$	$\mathrm{var}\left(g\right)={\mathbf{G}}_{g}=\mathbf{I}{\sigma }_{g}^{2}$	Identity
$gc$	$\mathrm{var}\left(gc\right)={\mathbf{G}}_{gc}=\mathbf{I}{\sigma }_{gc}^{2}$	Identity
$g(c)=g+gc$	$\mathrm{var}\left(g\left(c\right)\right)={\mathbf{G}}_{(g\left(c\right))}={\oplus }_{i=1}^{I}{\mathbf{G}}_{(g{\left(c\right))}_{i}} ,$	
	${\mathbf{G}}_{(g{\left(c\right))}_{i}}=\mathbf{I}{\sigma }_{gc}^{2}+\mathbf{J}{\sigma }_{g}^{2}$	Compound symmetry
	${\mathbf{G}}_{(g{\left(c\right))}_{i}}=\{{\sigma }_{{(g\left(c\right))}_{l{l}^{^{\prime}}}}^{2}\}$	Unstructured $l$ and ${l}^{^{\prime}}$ indicate countries
	${\mathbf{G}}_{(g{\left(c\right))}_{i}}=\left[{\varvec{\Lambda}}{{\varvec{\Lambda}}}^{T}\right]$	Factor analytic order 1 without diagonal variances
$y\left(c\right)=y+yc$	$\mathrm{var}\left(y\left(c\right)\right)={\mathbf{G}}_{y(c)}={\oplus }_{k=1}^{K}{\mathbf{G}}_{(y{\left(c\right))}_{k}}$, ${\mathbf{G}}_{(y{\left(c\right))}_{k}}=\{{\sigma }_{(y{\left(c\right))}_{l{l}^{^{\prime}}}}^{2}\}$	Unstructured $l$ and ${l}^{^{\prime}}$ indicate countries
$gy\left(c\right)=gy+gyc$	$\mathrm{var}\left(gy\left(c\right)\right)={\mathbf{G}}_{gy(c)}={\oplus }_{k=1}^{K}{\mathbf{G}}_{(gy{\left(c\right))}_{k}}$, ${\mathbf{G}}_{(gy{\left(c\right))}_{k}}=\{{\sigma }_{(g{y\left(c\right))}_{l{l}^{^{\prime}}}}^{2}\}$	Unstructured $l$ and ${l}^{^{\prime}}$ indicate countries
$s$	$\mathrm{var}\left(s\right)={\mathbf{G}}_{s}={\oplus }_{l=1}^{L}{\mathbf{G}}_{{s}_{jl}} ,$ ${\mathbf{G}}_{{s}_{j}}=\mathbf{I}{\sigma }_{{s}_{jl}}^{2}$	Heterogeneous country-specific
$sy$	$\mathrm{var}\left(sy\right)={\mathbf{G}}_{sy}={\oplus }_{l=1}^{L}{\mathbf{G}}_{({sy)}_{jlk}} ,$ ${\mathbf{G}}_{{(sy)}_{jlk}}=\mathbf{I}{\sigma }_{{(sy)}_{jlk}}^{2}$	Heterogeneous country-specific
$gs$	$\mathrm{var}\left(gs\right)={\mathbf{G}}_{gs}={\oplus }_{l=1}^{L}{\mathbf{G}}_{{(gs)}_{ijl}} ,$ ${\mathbf{G}}_{{(gs)}_{ijl}}=\mathbf{I}{\sigma }_{{(gs)}_{ijl}}^{2}$	Heterogeneous country-specific
$gsy$	$\mathrm{var}\left(gsy\right)={\mathbf{R}}_{gsy}={\oplus }_{l=1}^{L}{\mathbf{R}}_{l}$, ${\mathbf{R}}_{l}=\mathbf{I}{\sigma }_{l}^{2}$	Heterogeneous country-specific residual variance
${g}_{i}=({a}_{i}+{b}_{i}{x}_{1jl})$	$$\left[\begin{array}{c}{a}_{i}\\ {b}_{i}\end{array}\right]\sim iid N(0, {\mathbf{G}}_{{g}_{i}})$$ $${\mathbf{G}}_{{g}_{i}}=\left[\begin{array}{cc}{\sigma }_{a}^{2}& {\sigma }_{ab}\\ {\sigma }_{ab}& {\sigma }_{b}^{2}\end{array}\right]\otimes {\mathbf{I}}_{I}$$	Random coefficient regression for the genotype term
${(gc)}_{il}=({p}_{il}+{q}_{il}{x}_{1jl})$	$$\left[\begin{array}{c}{p}_{il}\\ {q}_{il}\end{array}\right]\sim iid N(0, {\mathbf{G}}_{({gc)}_{il}})$$ $${\mathbf{G}}_{({gc)}_{il}}=\left[\begin{array}{cc}{\sigma }_{p}^{2}& {\sigma }_{pq}\\ {\sigma }_{pq}& {\sigma }_{q}^{2}\end{array}\right]\otimes {\mathbf{I}}_{IL}$$	Random coefficient regression for the genotype-by-country term
${(g(c))}_{il}=({p}_{il}+{q}_{il}{x}_{1jl})$	$$\left[ {\begin{array}{*{20}c} {p_{{i\left( {Ger} \right)}} } \\ {p_{{i\left( {Pol} \right)}} } \\ {q_{{i\left( {Ger} \right)}} } \\ {q_{{i\left( {Pol} \right)}} } \\ \end{array} } \right] \sim MVN\left( {0, {\Sigma }_{reg} \oplus {\mathbf{G}}_{{c_{l} }} } \right)$$ $${\Sigma }_{reg}\otimes {\mathbf{G}}_{{c}_{l}}=\left[\begin{array}{cc}{\sigma }_{p}^{2}& {\sigma }_{pq}\\ {\sigma }_{pq}& {\sigma }_{q}^{2}\end{array}\right]\otimes {\mathbf{I}}_{I}\otimes \left[\begin{array}{cc}{\sigma }_{Ger}^{2}& {\sigma }_{GerPol}\\ {\sigma }_{GerPol}& {\sigma }_{Pol}^{2}\end{array}\right]$$	Random coefficient regression for the genotype and genotype-by-country terms

The index *i* refers to the *i*th genotype, *j* refers to the *j*th location, and *l* refers to the *l*th country or zone. The country term, $c$, is replaced with $z$ for the agroecological zone level analysis. The symbol $\otimes$ represents a Kronecker product of matrices, while symbol $\oplus$ represents a direct sum of matrices

## Genetic and non-genetic trend analysis and incorporation of nitrogen application as a covariate in the country level analysis

Model 2 can be augmented for conducting genetic and non-genetic trend analysis as demonstrated by Piepho et al. (2014), Laidig et al. (2014) and Hadasch et al. (2020). The inclusion of covariates for nitrogen application and year of trial allows a correction for non-genetic trend. Equally importantly, fitting a covariate for the first trial year of a variety allows the estimation of genetic correlation not overly driven by large variation across years generated by genetic gain in the trial period, but more driven by the genetic correlation of common varieties released in a single year. The extended model is

4 $${yield}_{ijlk}=\mu +{c}_{l}+{\beta }_{1}{x}_{1jl}+{\eta }_{l}{x}_{1jl}+{\beta }_{2}{x}_{2i}+{\beta }_{3}{x}_{3i}+{g}_{i}+{(gc)}_{il}+{y}_{k}+{(yc)}_{kl}+{(gy)}_{ik}+ {(gyc)}_{ikl}+{s}_{jl}+ {(sy)}_{jlk}+{(gs)}_{ijl}+{(gsy)}_{ijlk}^{^{\prime}}$$

where ${x}_{1jl}$ is location-specific nitrogen application, ${x}_{2i}$ is the first trial year of a variety, ${x}_{3i}$ is the calendar year of testing of a variety. The notation for fixed regression terms involving the covariate is ${\beta }_{1}{x}_{1jl}+{\eta }_{l}{x}_{1jl}+{\beta }_{2}{x}_{2i}+{\beta }_{3}{x}_{3i}$, where ${\beta }_{1}$, ${\beta }_{2}$ and ${\beta }_{3}$ are the fixed effects for the slopes of nitrogen application, first trial year of a variety and calendar year of testing of a variety, whereas ${\eta }_{l}$ is the country-specific slope of the nitrogen application of the *l*th country. The nitrogen covariate was mean-centred and then divided by 500, since this scaling resulted in non-negative variance estimates for the random coefficient models. The first year of testing and calendar year were mean-centred.

The fixed and random effects genotype models are used in Model 4. The FGC is Model 4 with fixed genotype effects, and the RGC is Model 4 with random genotype effects. For the random genotype effects, the model can be extended to a random coefficient model, which includes the interaction of genotype × nitrogen application and genotype × country × nitrogen application. In this paper, four random coefficient models are fitted. RC1 is a model with random coefficients. A random coefficients model was fitted for the genotype term, ${g}_{i}$, and the genotype × country term, $({gc})_{il}$. Hence, this model has genotype and genotype × country-specific coefficients for the intercepts and slopes. RC2 is a reduced model of RC1, where the random regression coefficients in the genotype main effect are dropped. RC3 is another modification of RC1, in which the random regression coefficients in the genotype × country effect are dropped. RC4 is a modification of RC1 with random regression coefficients for the genotype and genotype-by-country terms with ${\Sigma }_{reg}\otimes {\mathbf{G}}_{{c}_{l}}$ variance–covariance structure (where symbol $\otimes$ represents the Kroneker product of matrices). Thus, the variance structure of the intercepts and slopes is country-specific and allows for covariances between the slopes and intercepts for each of the countries. The details of variance–covariance structures for random coefficient models are explained in the next section and summarised in Table 4. All models were fitted in R (R Core Team, 2022) using ASReml-R package version 4.1.0.130 (Butler et al. 2017).

## Variance–covariance structures in relation to the random effects of genotype

Genotypes in trials can be regarded as a random sample from a population of genotypes and to exploit the genetic correlation between countries and to extend the variance–covariance structure implied by the Model 2, we merged the genetic main effect ${g}_{i}$ and the genotype × country interaction ${(gc)}_{il}$ into a composite genetic effect ${(g\left(c\right))}_{il}$ nested within countries, that is,

5 $$\left( {g\left( c \right)} \right)_{il} = g_{i} + \left( {gc} \right)_{il}$$

If we consider agroecological zones to exploit the genetic correlation between zones, the factor ($c$) is replaced by the factor ($z$).

The variance–covariance structure in Eq. 5 is based on the common genotypes in both countries, i.e. $({g}_{i\left(ger\right)}, {g}_{i\left(pol\right)})$, and can have different structures. The compound symmetry (CS) structure in Eq. 6 is implied by Model 2 if both ${g}_{i}$ and ${(gc)}_{il}$ have constant variance. The term ${\sigma }_{g}^{2}+{\sigma }_{gc}^{2}$ on the diagonal of the matrix in Eq. 6 is the variance of genotypes within one country, and the covariance of the same genotype between countries is ${\sigma }_{g}^{2}$, which is on the off-diagonal of the matrix.

6 $$\left[\begin{array}{cc}{\sigma }_{g}^{2}+{\sigma }_{gc}^{2}& {\sigma }_{g}^{2}\\ {\sigma }_{g}^{2}& {\sigma }_{g}^{2}+{\sigma }_{gc}^{2}\end{array}\right]$$

The CS structure assumes a common variance within countries and a common covariance between countries, which is very restrictive and, in many cases, is an unrealistic assumption. The unstructured (UN) variance–covariance given in Eq. 7 is more flexible than the CS structure in the sense that it allows different variances and a separate parameter ${\rho }_{l{l}^{^{\prime}}}$ that specifies the correlation between countries *l* and ${l}^{^{\prime}}$.

7 $$\left[\begin{array}{cc}{\sigma }_{g(l)}^{2}& {\sigma }_{g(l)}{\sigma }_{g({l}^{^{\prime}})}{\rho }_{{ll}^{^{\prime}}}\\ {\sigma }_{g(l)}{\sigma }_{g({l}^{^{\prime}})}{\rho }_{{ll}^{^{\prime}}}& {\sigma }_{g({l}^{^{\prime}})}^{2}\end{array}\right]$$

In the case of two countries, the UN structure is easy to fit. However, this variance–covariance structure becomes very complex if there are several countries or agroecological zones. For example, with five zones, 15 variance–covariance component estimates need to be estimated in the unstructured model. A less computationally expensive structure allowing for heterogeneity of variances and covariances is the factor analytic (FA) model. The first-order FA (FA1) model is composed of covariance terms that are defined as the product ${\lambda }_{l}{\lambda }_{{l}^{^{\prime}}}$, where ${\lambda }_{l}$ is the loading for the *l*th country/zone. The variance for the *l*th country/zones is represented by the term ${\lambda }_{l}^{2}$ and an additional variance component ${\psi }_{l}$. It is also possible to have an FA structure by omitting the term ${\psi }_{l}$, which here is called reduced rank FA or FA-0. The FA1 structure for five zones needs only five ${\lambda }_{l}$ terms and five ${\psi }_{l}$ terms as shown in Eq. 8, which is less expensive than the UN structure. When Eq. 8 uses the reduced rank FA order 1 structure (FA-01), then it needs only five ${\lambda }_{l}$ terms.

8 $${\varvec{\Lambda}}{{\varvec{\Lambda}}}^{\mathrm{T}}+{\varvec{\Psi}}=\left[\begin{array}{ccccc}{\lambda }_{1}^{2}+{\psi }_{1}& {\lambda }_{1}{\lambda }_{2}& {\lambda }_{1}{\lambda }_{3}& {\lambda }_{1}{\lambda }_{4}& {\lambda }_{1}{\lambda }_{5}\\ {\lambda }_{2}{\lambda }_{1}& {\lambda }_{2}^{2}+{\psi }_{2}& {\lambda }_{2}{\lambda }_{3}& {\lambda }_{2}{\lambda }_{4}& {\lambda }_{2}{\lambda }_{5}\\ {\lambda }_{3}{\lambda }_{1}& {\lambda }_{3}{\lambda }_{2}& {\lambda }_{3}^{2}+{\psi }_{3}& {\lambda }_{3}{\lambda }_{4}& {\lambda }_{3}{\lambda }_{5}\\ {\lambda }_{4}{\lambda }_{1}& {\lambda }_{4}{\lambda }_{2}& {\lambda }_{4}{\lambda }_{3}& {\lambda }_{4}^{2}+{\psi }_{4}& {\lambda }_{4}{\lambda }_{5}\\ {\lambda }_{5}{\lambda }_{1}& {\lambda }_{5}{\lambda }_{2}& {\lambda }_{5}{\lambda }_{3}& {\lambda }_{5}{\lambda }_{4}& {\lambda }_{5}^{2}+{\psi }_{5}\end{array}\right]$$

In Model 4, we include the nitrogen application as a covariate. Thus, when the genotype effect is random, the genotype-specific regression must be modelled as random effects as well, as demonstrated by Buntaran et al. (2021). Therefore, the term ${g}_{i}$ can be expanded as a random coefficient of genotype × nitrogen application, ${g}_{i}={a}_{i}+{b}_{i}{x}_{1jl}$, where ${a}_{i}$ is the random intercept for the $i$ th genotype and ${b}_{i}$ is the random slope for the $i$ th genotype. In this case, the variance–covariance structure of ${\mathbf{G}}_{g}$ has variance estimates for the random intercept and random slopes with a covariance between random intercept and random slope. Furthermore, in the random coefficient model, it is important to have the UN structure for ${\mathbf{G}}_{g}$ to ensure invariance with respect to translation and scale transformation of the covariate (Longford, 1993; Wolfinger, 1996; Piepho and Ogutu, 2002). The random coefficient can also be applied for the genotype × country × nitrogen application term, which is expanded as ${(gc)}_{il}={p}_{il}+{q}_{il}{x}_{1jl}$, where ${p}_{il}$ is the random intercept for the $i$ th genotype in the $l$ th country and ${q}_{il}$ is the random slope for the $i$ th genotype in the $l$ th country.

## Results

### Country level analysis

The fit statistics for all models listed in Table 3 are reported in Table 5. The Akaike information criterion (AIC) based on the full maximum likelihood method is used to compare different fixed effects terms in the models, while the AIC based on the restricted maximum likelihood (REML) was used to compare models with different variance–covariance structures for the random effects. For all models, the AIC was calculated (the smaller AIC is a better fit) using the infoCriteria function from the asremlPlus library.

**Table 5:** Fit statistics for model selection of eight models fitted with (restricted) maximum likelihood method from country-based analysis

Model	Variance structure for $gc$	No. of fixed effect terms	No. of Covariance parameters*	Restricted maximum likelihood		Full maximum likelihood	
				Log- likelihood	AIC	Log-likelihood	AIC
FG**		3	14	− 72,033	144,094	–	–
FGC		7	14	− 71,894	143,816	–	–
RG﻿†							
	CS	2	15	− 74,143	148,317	− 74,145	148,325
	FA-01	2	16	− 74,143	148,319	− 74,145	148,327
	US	2	16	− 74,143	148,319	− 74,145	148,327
RGC							
	CS	6	16	− 73,865	147,762	− 73,866	147,776
	FA-01	6	16	− 73,956	147,944	− 73,957	147,958
	US	6	17	− 73,863	147,759	− 73,864	147,773
RC1		6	19	− 73,862	147,759	− 73,863	147,773
RC2		6	17	− 73,862	147,759	− 73,863	147,773
RC3		6	17	− 73,863	147,761	− 73,864	147,775
RC4		6	18	− 73,860	147,757	− 73,861	147,771

*Non-bounded parameters

**Baseline models for fixed genotype models

^†^Baseline model for random genotype models

The infoCriteria function could not compute the full likelihood of FG and FGC models (as the iterations did not converge), so we could not compare these two models. Furthermore, the main purpose of the analysis is to borrow strength between countries, so the comparison of the RG and RGC models is more essential. The complex variance–covariance structure in the RG model did not improve the fit statistics since the AIC based on REML was slightly higher for FA-01 and UN structures than for CS. Therefore, the simpler CS structure was sufficient to explain the variations of $gc$ interaction effects.

For the RGC model, the UN structure had the smallest REML-based AIC, although the value was only slightly smaller than that for the CS structure. On the other hand, the FA-01 structure had a far larger AIC compared to the UN and CS structures. Compared to the RG model, the RGC model had a smaller AIC-full maximum likelihood based on the same variance–covariance structure for $gc$. Thus, the covariates improved the fit statistics. Among the random coefficient models, the RC4 model had the smallest AIC based on both REML and full maximum likelihood. The RC4 model fitted slightly differently from the other random coefficient models because it had the ${\Sigma }_{reg}\otimes {\mathbf{G}}_{{c}_{l}}$ structure as shown in Table 4, which combined the random coefficient regression for the $g$ and $gc$ terms in a single term $g\left(c\right)$. The AIC between RC1 and RC2 were the same, which showed that dropping the random coefficient term in the genotype did not change the fit statistics. However, when the random coefficient was only retained for the genotype term, the AIC increased.

Table 6 presents the variance component estimates for model RGC-UN and RC4, with the smallest AIC (estimates of variance components from all models are given in the supplementary Table S1). In these two models, the genetic correlations between the two countries, ${\rho }_{g({c}_{\mathrm{1,2}})}$, were very similar, i.e. 0.890 and 0.884, for the RGC-UN and the RC4 models, respectively. This suggests that the performance and the rank of the overlapping genotypes between the two countries were quite similar. Moreover, the genetic and non-genetic trends and the nitrogen application effects were similar for the two models. Both the regression coefficient of the nitrogen application and the genetic trend were positive. The regression coefficient for non-genetic trend was negative but non-significant. However, the genetic trend and nitrogen application coefficients were positive and larger than the non-genetic trend, so overall the yield was still increasing.

**Table 6:** Estimates of covariates and variance components of two best model (RGC and RC4) and associated standard errors (s.e.)

	RGC-UN		RC4	
	Estimate	s.e	Estimate	s.e
Fixed﻿ effects				
Nitrogen $({\beta }_{1})$	14.7611*	2.9224	14.9681*	3.0072
Genetic $({\beta }_{2})$	1.4695*	0.0552	1.4686*	0.0549
Non-genetic $({\beta }_{3})$	− 0.2882^ ns^	0.1831	− 0.2876^ ns^	0.1830
Random effects^†^				
*Genotype× country*				
${\sigma }_{g{(c}_{1})}^{2}$	11.292	1.144	10.067	–
${\sigma }_{g({c}_{2})}^{2}$	15.021	1.313	13.834	–
${\rho }_{g({c}_{\mathrm{1,2}})}$	0.890	0.086	0.884	–
${\sigma }_{{q}_{1}}^{2}$	–	–	18.273	–
${\sigma }_{{q}_{2}}^{2}$	–	–	25.113	–
${\rho }_{(p, {q)}_{1}}$	–	–	− 0.133	–
${\rho }_{(p{,q}_{)2}}$	–	–	− 0.133	–
*Year* × *country*				
${\sigma }_{y({c}_{1})}^{2}$	59.706	17.728	59.677	17.720
${\sigma }_{y{(c}_{2})}^{2}$	191.284	57.894	191.241	57.880
${\rho }_{y{(c}_{\mathrm{1,2}})}$	0.812	0.091	0.812	0.091
*Genotype* × *year* × *country*				
${\sigma }_{gy({c}_{1})}^{2}$	5.144	0.451	5.135	0.451
${\sigma }_{gy{(c}_{2})}^{2}$	6.269	0.620	6.216	0.614
${\rho }_{gy({c}_{\mathrm{1,2}})}$	0.126	0.339	0.121	0.340
*Location* × *country*				
${\sigma }_{s({c}_{1})}^{2}$	99.976	19.778	99.948	19.774
${\sigma }_{s({c}_{2})}^{2}$	89.923	28.910	89.908	28.906
*Year* × *location* × *country*				
${\sigma }_{sy({c}_{1})}^{2}$	155.660	8.899	155.681	8.900
${\sigma }_{sy({c}_{2})}^{2}$	172.154	13.036	172.145	13.036
*Genotype* × *location* × *country*				
${\sigma }_{gs({c}_{1})}^{2}$	4.356	0.412	4.299	0.413
${\sigma }_{gs({c}_{2})}^{2}$	8.462	0.494	8.445	0.494
*Residual*^*‡*^				
${\sigma }_{{\varepsilon }_{1}}^{2}$	27.775	0.475	27.745	0.476
${\sigma }_{{\varepsilon }_{2}}^{2}$	27.359	0.486	27.313	0.485

Significance level: *P* < 0.05 (*) and not-significant (ns)

^†^The subscript in random effects “1” represents Germany and “2” represents Poland

^‡^The residual is the sum of the three-way interaction $(gsy)$ and the residual

**Fig. 4:**
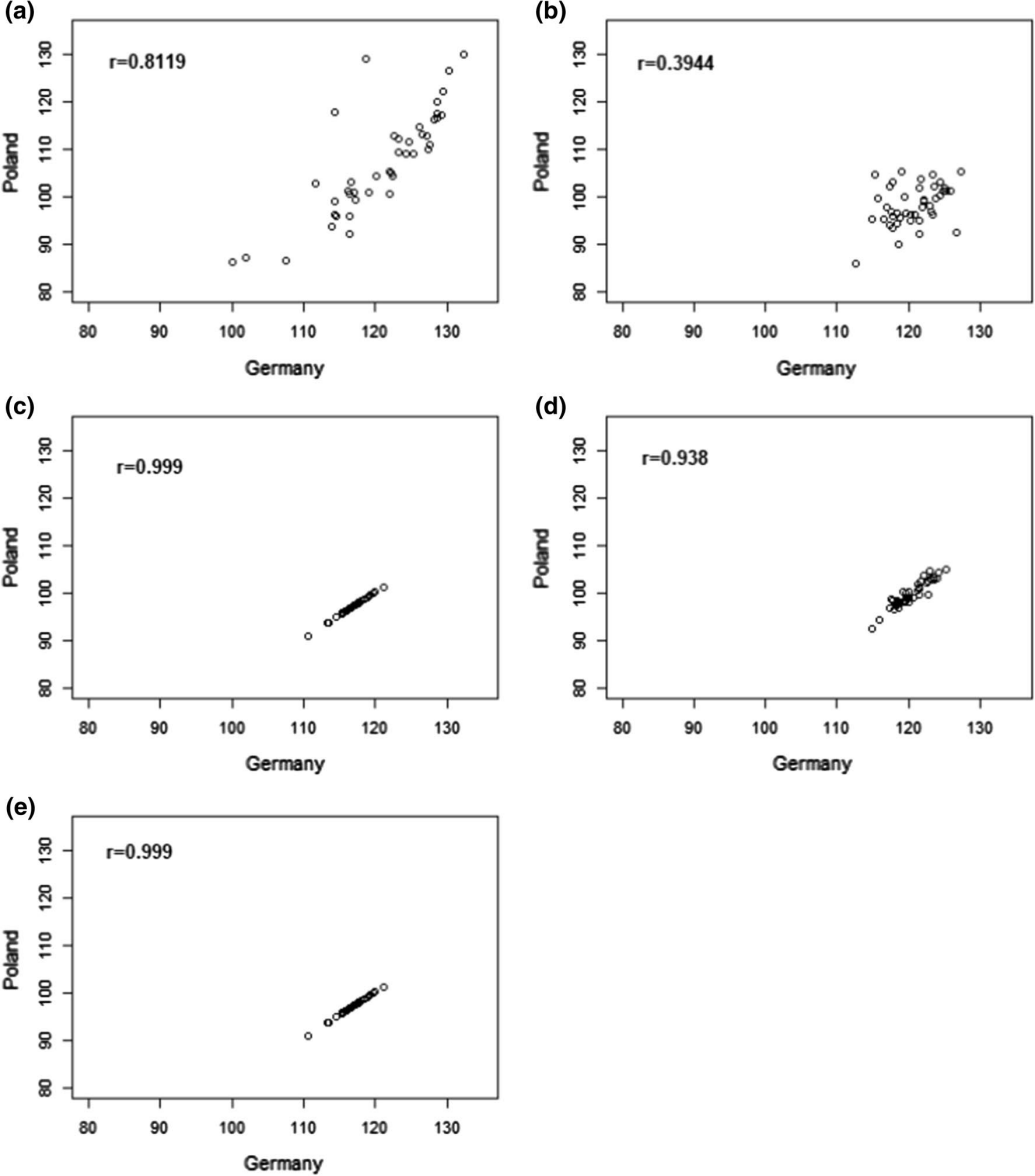
Yield prediction of 43 common varieties in Germany and Poland from **a** FG, **b** FGC, **c** RG-UN, **d** RGC-UN and **e** RC4 models

Figure 4 depicts the country pair-wise scatterplots of genotype estimates of genotye × country interaction effects from the FG, FGC, RG-UN, RGC-UN and RC4 models. There is a clear distinction between the fixed genotype effect models (FG and FGC) and the random genotype effect models (RGs and RC models). The estimates in the random genotype effect models were more similar between the two countries compared to the fixed genotype effect models, as a result of borrowing information, i.e. the genetic correlation exploited between the two countries. Moreover, we can see that the correlations between mean yield from the two countries from random genotype effect models were close to 1. The high correlation implies that the performance and ranking of the common genotypes were very similar between Germany and Poland.

### Agroecological zone level analysis

The zone-based analysis per country was performed prior to the joint analysis of agroecological zones between countries which examines the correlations between zones within a country. The analysis was performed using the RGC model and replacing country effect (${c}_{l}$) with zone effect (${z}_{l}$). The estimates of variance components from the analysis are given in Table 7. The variance estimate of the genotype × zone effect in Poland was zero, which means that there is no necessity for agroecological zonation or division for maize. However, a small variation in genotype × zone effect can be seen in the German data. The estimates of genotype variance in each zone and zone correlations for German data are given in supplementary Table S2. The small genotype × zone variance estimate in Table 7 for both countries can be explained by the fact that the genotype variance estimates were very similar across zones and there were strong correlations between zones.

**Table 7:** Estimates of variance components and their standard errors of using RGC model for agroecological zones in Poland and Germany

Effect	Poland			Germany		
	Zone	Estimate	s.e	Zone	Estimate	s.e
Genotype		19.38	1.29		13.94	1.22
Genotype × zone		0.00	NA		0.25	0.27
Year × zone	P:1	196.33	66.21	D:1	87.97	30.77
	P:2	258.45	93.68	D:2	72.46	23.47
	P:3	271.25	94.73	D:3	91.09	32.74
	P:4	49.80	34.47	D:4	40.99	32.14
				D:5	10.79	30.39
Location × zone	P:1	68.10	37.93	D:1	47.85	23.76
	P:2	114.26	76.99	D:2	76.78	28.55
	P:3	35.32	31.48	D:3	7.91	11.41
	P:4	97.64	91.91	D:4	30.14	26.75
				D:5	218.69	117.52
Year × location × zone	P:1	140.17	18.66	D:1	104.23	14.29
	P:2	226.10	36.37	D:2	131.49	12.56
	P:3	140.61	27.50	D:3	140.95	20.26
	P:4	152.76	35.24	D:4	239.96	41.03
Genotype × year × zone				D:5	188.94	46.12
	P:1	4.29	0.55	D:1	6.16	0.84
	P:2	7.01	0.80	D:2	6.31	0.63
	P:3	5.08	0.79	D:3	6.39	0.95
	P:4	8.63	1.32	D:4	6.63	1.16
				D:5	1.89	0.99
Genotype × location × zone	P:1	7.57	0.82	D:1	1.83	0.73
	P:2	6.48	0.83	D:2	4.69	0.62
	P:3	9.36	1.12	D:3	2.11	1.00
	P:4	6.23	1.27	D:4	2.55	1.30
				D:5	3.38	1.35

As there were not enough genotypes tested for several years across zones in the German data to fit more complex models, the heterogeneous diagonal zone-specific variance structure for genotype × year × zone effect was used. By comparison, in the Polish data, the unstructured variance could be fitted for the genotype × year × zone effect. However, to be consistent for both countries, we also fitted the heterogeneous diagonal zone-specific structure for the genotype × year × zone effect in the Polish data.

**Fig. 5:**
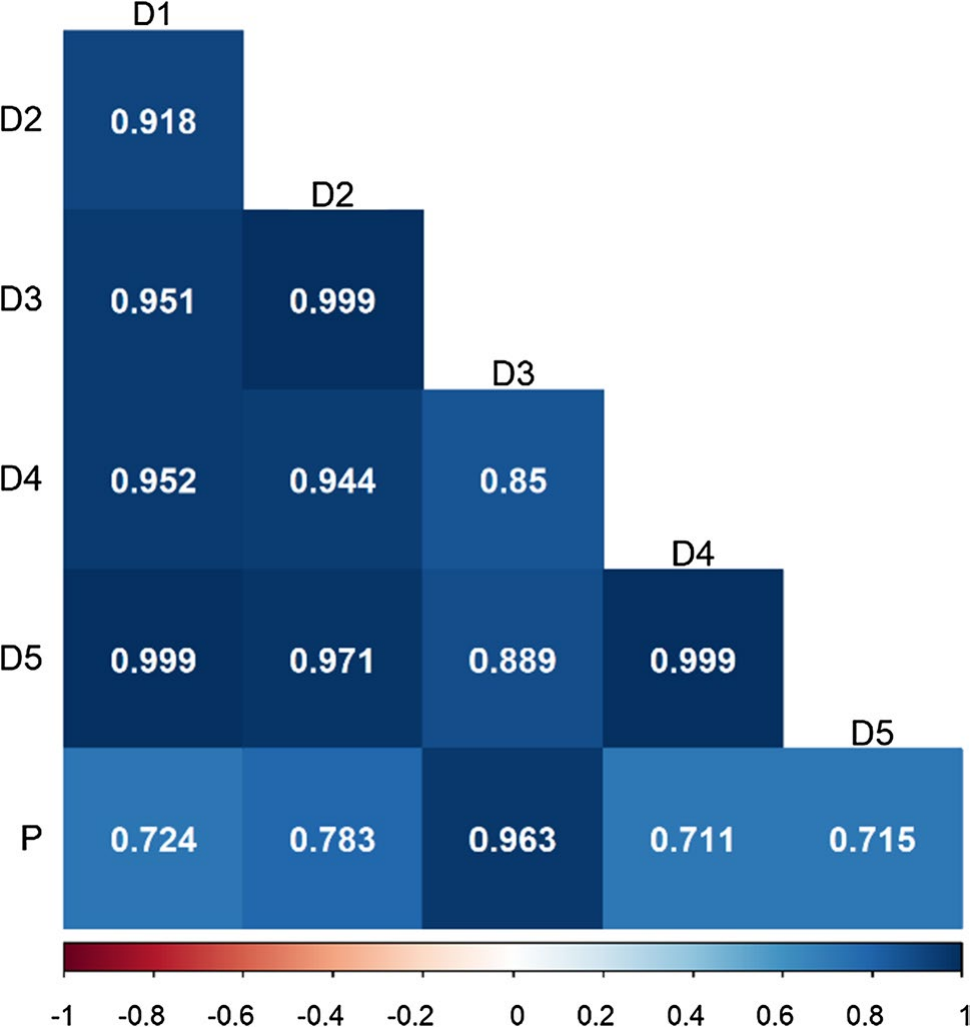
Genetic correlations between German zones and Poland using RGC model

Since the variance estimate of genotype × zone effects in the Polish data was zero, in the joint analysis of German and Polish agroecological zones, we merged all zones of Poland into one zone. Thus, the dataset for the joint analysis consisted of five zones in Germany and Poland as one zone. The analysis based on agroecological zones was performed using the RGC model. The estimates of variance components from zone-based analysis using the RGC model are given in supplementary Table S3. The genetic correlations between German zones and Poland are given in Fig. 5. In most of the cases, the genetic correlations are high between the German zones; however, only German zone D3 has a high correlation with Poland. The genetic correlation between zones was based on the performance of the common genotypes. Although the zones are far from each other on the map, the performances of the common genotypes between these zones were very similar, making the genetic correlation high.

## Discussion

A high genetic correlation was observed between Germany and Poland (Table 6), and this is reflected in the similarity of genetic ranking based on the mean yield of common varieties from Germany and Poland reported in Fig. 4. The fitting of heteroscedastic variance–covariance structures to the genotype × country classification provides a view of the similarities between countries. The fit in terms of AIC was not improved for the heterogeneous UN model; however, higher-order models should be preferred to avoid underfitting and the resulting bias (Piepho, 2008). The incorporation of covariates allows for improving models as given in Table 5. The random coefficient models enable the user to fit genotype and genotype × country regressions. However, the results show that random coefficient models could not improve the fit because of the high similarity between Germany and Poland, and genotypes performing similarly in both countries. Because of the high genetic correlation between Germany and Poland, one country can benefit from using maize trial data from the other country and vice versa.

The stratification of locations into zones and the use of random effects models for the genotype × zone classification allows for the borrowing of strength across zones when estimating mean yield per zone. However, to attain reliable estimates of genotype effects, it is necessary for the zone to be represented by a suitable number of locations (Kleinknecht et al. 2013). Many zones in Germany and Poland were not represented by enough locations (Fig. 3); therefore, these zones were merged with the neighbouring zones after assessing the genetic correlations between zones. This resulted in five zones in Germany and four zones in Poland, subsequently used for zone-specific analysis. The heteroscedastic variance–covariance structures used for the genotype × zone classification provided estimates of similarities between zones. The variance estimate of the genotype × zone effect in Poland was zero (Table 7), which means that there is no necessity for agroecological zonation or division for maize. However, a small variation in the genotype × zone effect can be seen in German data. The estimates of genetic correlation between Germany and Poland zones (Fig. 5) indicate that the German zone D3 is highly correlated with Poland. The German zones are also very similar to one another in terms of the mean yield comparisons among genotypes. The high genetic correlation between German and Polish zones does not necessarily mean that zones are agroecologically similar, although climatic and soil conditions in these zones are distinct from one another (Graf et al. 2009). It is well possible that the mean yield of genotypes in the zones are not significantly affected by agroecological differences among the zones, so a high genetic correlation can occur despite such differences, especially when the genetic variance is not very large compared to variance components for genotype × environment interaction effects.

## Supplementary Information

Below is the link to the electronic supplementary material.

Supplementary file1 (DOCX 46 kb)

## Data Availability

Data were provided by the Federal Plant Variety Office Germany and Research Centre for Cultivar Testing Poland for exclusive use in this study and are in general not publicly available. Reasonable requests may be addressed to Federal Plant Variety Office, Hannover, Germany, and Research Centre for Cultivar Testing, Słupia Wielka, Poland.

## References

[CR1] Buntaran H, Piepho HP, Hagman J, Forkman J (2019). A cross-validation of statistical models for zoned-based prediction in cultivar testing. Crop Sci.

[CR2] Buntaran H, Piepho HP, Schmidt P, Rydén J, Halling M, Forkman J (2020). Cross-validation of stage-wise mixed-model analysis of Swedish variety trials with winter wheat and spring barley. Crop Sci.

[CR3] Buntaran H, Forkman J, Piepho HP (2021). Projecting results of zoned multi-environment trials to new locations using environmental covariates with random coefficient models: accuracy and precision. Theor Appl Genet.

[CR4] Butler DG, Cullis B, Gilmour A, Gogel BJ, Thompson R (2017). ASReml-R reference manual, version 4.

[CR5] Denis JB (1988). Two-way analysis using covariates. Statistics.

[CR6] Gauch HG, Zobel RW (1997). Identifying mega-environments and targeting genotypes. Crop Sc.

[CR7] Graf R, Michel V, Roßberg D, Neukampf R (2009). Definition pflanzenartspezifischer Anbaugebiete für ein regionalisiertes Versuchswesen im Pflanzenbau. J Für Kulturpflanzen.

[CR8] Hadasch S, Laidig F, Macholdt J, Bönecke E, Piepho HP (2020). Trends in mean performance and stability of winter wheat and winter rye yields in a long-term series of variety trials. Field Crops Res.

[CR9] Kleinknecht K, Möhring J, Singh KP, Zaidi PH, Atlin GN, Piepho HP (2013). Comparison of the performance of best linear unbiased estimation and best linear unbiased prediction of genotype effects from zoned Indian maize data. Crop Sci.

[CR10] Laidig F, Drobek T, Meyer U (2008). Genotypic and environmental variability of yield for cultivars from 30 different crops in German official variety trials. Plant Breed.

[CR11] Laidig F, Piepho HP, Drobek T, Meyer U (2014). Genetic and non-genetic long-term trends of 12 different crops in German official variety performance trials and on-farm yield trends. Theor Appl Genet.

[CR12] Longford NT (1993). Random coefficient models.

[CR13] Maiskomitee D (2022) Deutsches Maiskomitee e.V. (DMK). www.maiskomitee.de

[CR14] Piepho HP, Möhring J (2005). Best linear unbiased prediction of cultivar effects for subdivided target regions. Crop Sci.

[CR15] Piepho HP, Ogutu JO (2002). A simple mixed model for trend analysis in wildlife populations. J Agric Biol Environ Stat.

[CR16] Piepho HP, Möhring J, Melchinger AE, Büchse A (2008). BLUP for phenotypic selection in plant breeding and variety selection. Euphytica.

[CR17] Piepho HP, Laidig F, Drobek T, Meyer U (2014). Dissecting genetic and non-genetic sources of long-term yield trend in German official variety trials. Theor Appl Genet.

[CR18] R Core Team (2022) R: a language and environment for statistical computing. R Foundation for Statistical Computing, Vienna, Austria. https://www.R-project.org

[CR19] van Eeuwijk FA, Bustos-Korts DV, Malosetti M (2016). What should students in plant breeding know about the statistical aspects of genotype × environment interactions?. Crop Sci.

[CR20] Wolfinger RD (1996). Heterogeneous variance: covariance structures for repeated measures. J Agric Biol Environ Stat.

