# Supplementary material for: Assessing the between-country genetic correlation in maize yield using German and Polish official variety trials
Source: Theor Appl Genet. 2022 Jul 13;135(9):3025–38. doi: 10.1007/s00122-022-04164-2 (PMC9482609; doi:10.1007/s00122-022-04164-2)
Supplement: Supplementary file 1 — Supplementary file1 (DOCX 46 kb) [file 122_2022_4164_MOESM1_ESM.docx]

# **Supplementary**

**Table S1** Estimates of variance components from all models.

|  | | | Random Genotype | | | Random Genotypes with covariates (First year of testing, Trial year, Nitrogen, Nitrogen:Country) | | | Random Genotypes with Random coefficient regression (First year of testing, Trial year, Nitrogen, Nitrogen:Country) | | | |
| --- | --- | --- | --- | --- | --- | --- | --- | --- | --- | --- | --- | --- |
|  | Factor | Group | RG-CS | RG-FA01 | RG-UN | RGC1-CS | RGC1-FA01 | RGC1-UN | RC1 | RC2 | RC3 | RC4* |
| Intercept | G |  | 9.343 |  |  | 13.280 |  |  | - | 11.783 | - |  |
| Intercept | G:C | DE |  | 3.070 | 9.423 |  | 3.061 | 11.292 | - | - | 1.562 |  |
| Intercept | G:C | PL |  | 3.044 | 9.267 |  | -3.472 | 15.021 | - | - | - |  |
| Cor(DE, PL) | G:C |  | 0.999 |  | 0.999 | 0.880 |  | 0.890 | - | - | - |  |
| Corr(Int, Slope) | G |  | - | - | - | - | - | - | 0.999 | - | -0.161 |  |
| Intercept | G |  | - | - | - | - | - | - | 11.783 | - | 11.719 |  |
| Slope | G |  | - | - | - | - | - | - | 0.000 | - | 15.655 |  |
| Corr(Int, Slope) | G:C |  | - | - | - | - | - | - | -0.570 | -0.569 | - |  |
| Intercept | G:C |  | - | - | - | - | - | - | 1.509 | 1.508 | - |  |
| Slope | G:C |  | - | - | - | - | - | - | 18.200 | 18.201 | - |  |
| Cor(DE, PL) | Y:C |  | 0.870 | 0.870 | 0.870 | 0.813 | 0.807 | 0.812 | 0.813 | 0.813 | 0.813 | 0.812 |
| Intercept | Y:C | DE | 169.509 | 169.383 | 169.383 | 59.609 | 61.374 | 59.706 | 59.564 | 59.564 | 59.576 | 59.677 |
|  |  | PL | 310.152 | 310.041 | 310.074 | 191.289 | 191.034 | 191.284 | 191.262 | 191.262 | 191.240 | 191.241 |
| Cor(DE, PL) | G:Y:C |  | -0.131 | -0.134 | -0.134 | 0.091 | 0.444 | 0.126 | 0.140 | 0.140 | 0.082 | 0.121 |
| Intercept | G:Y:C | DE | 12.481 | 12.461 | 12.461 | 5.007 | 7.117 | 5.144 | 4.983 | 4.983 | 4.984 | 5.135 |
|  |  | PL | 11.186 | 11.217 | 11.217 | 6.609 | 9.167 | 6.269 | 6.580 | 6.580 | 6.592 | 6.216 |
| Intercept | L:C | DE | 104.352 | 104.354 | 104.355 | 100.025 | 99.745 | 99.976 | 100.032 | 100.032 | 100.009 | 99.948 |
|  |  | PL | 88.631 | 88.631 | 88.631 | 89.929 | 90.180 | 89.923 | 89.925 | 89.925 | 89.916 | 89.908 |
| Intercept | Y:L:C | DE | 155.992 | 155.991 | 155.991 | 155.651 | 155.749 | 155.660 | 155.665 | 155.665 | 155.668 | 155.681 |
|  |  | PL | 172.753 | 172.753 | 172.753 | 172.152 | 172.105 | 172.154 | 172.151 | 172.151 | 172.144 | 172.145 |
| Intercept | G:L:C | DE | 4.322 | 4.321 | 4.321 | 4.350 | 4.365 | 4.356 | 4.294 | 4.294 | 4.299 | 4.299 |
|  |  | PL | 8.474 | 8.475 | 8.475 | 8.482 | 8.619 | 8.462 | 8.471 | 8.471 | 8.472 | 8.445 |
| Residual |  | DE | 27.830 | 27.831 | 27.831 | 27.778 | 27.753 | 27.775 | 27.747 | 27.747 | 27.751 | 27.745 |
|  |  | PL | 27.346 | 27.345 | 27.345 | 27.348 | 27.261 | 27.359 | 27.310 | 27.310 | 27.318 | 27.313 |
| * See below for computation of correlations from RC4 model. | | | | | | | | | | | | |

|  | Variance-Covariance ($\Sigma_{reg})$ | |  |  | Variance-Covariance ($\mathbf{G}_{c_{l}}$) | |
| --- | --- | --- | --- | --- | --- | --- |
|  | int | slope |  |  | GER | POL |
| int | 1.1000 | -0.1873 |  | GER | 10.0667 | 10.4304 |
| slope | -0.1873 | 1.8152 |  | PL | 10.4304 | 13.8345 |

$$\Sigma_{reg}\otimes\mathbf{G}_{c_{l}}=\left[ \begin{matrix} \sigma_{p}^{2} & \sigma_{pq} \\ \sigma_{pq} & \sigma_{q}^{2} \end{matrix} \right]\otimes\mathbf{I}_{I}\otimes\left[ \begin{matrix} \sigma_{Ger}^{2} & \sigma_{GerPol} \\ \sigma_{GerPol} & \sigma_{Pol}^{2} \end{matrix} \right]$$

| Variance-Covariance ($\Sigma_{reg}\otimes\mathbf{G}_{c_{l}})$ | | | | |
| --- | --- | --- | --- | --- |
|  | int_ GER | int_POL | slope_ GER | slope_POL |
| int_ GER | 11.073 | 11.473 |  |  |
| int_ POL | 11.473 | 15.218 |  |  |
| slope_ GER | -1.885 | -1.954 | 18.273 |  |
| slope_ POL | -1.954 | -2.591 | 18.933 | 25.113 |

Correlation between Germany and Poland based on the above variance-covariance matrix ($\rho_{g(c_{1,2})}$) = 0.884

**Table S2a** Estimates of variance components of genotype×zone effects and their associated standard errors (s.e.) from the analysis of German agroecological zones.

| Effect | Zone | Estimate | s.e. |
| --- | --- | --- | --- |
| Genotype×zone | D:1 | 16.388 | 1.793 |
|  | D:2 | 12.809 | 1.382 |
|  | D:3 | 13.480 | 1.586 |
|  | D:4 | 16.260 | 1.952 |
|  | D:5 | 15.473 | 1.857 |

**Table S2b** Estimated correlations and their standard errors (in parentheses) of genotype×zone effects from the analysis of German agroecological zones.

|  | D1 | D2 | D3 | D4 | D5 |
| --- | --- | --- | --- | --- | --- |
| D1 |  |  |  |  |  |
| D2 | 0.921  (0.035) |  |  |  |  |
| D3 | 0.961 (0.961) | 0.999 (NA) |  |  |  |
| D4 | 0.953 (0.035) | 0.945 (0.035) | 0.854 (0.047) |  |  |
| D5 | 0.999 (NA) | 0.971 (0.035) | 0.899 (0.043) | 0.999 (NA |  |

**Table S3a** Estimates of variance components and associated standard errors (s.e.) from agroecological zone based analysis.

| Effect | Zone | Estimate | s.e. |
| --- | --- | --- | --- |
| Genotype×zone | D:1 | 15.98 | 1.75 |
|  | D:2 | 12.48 | 1.34 |
|  | D:3 | 13.09 | 1.51 |
|  | D:4 | 15.83 | 1.91 |
|  | D:5 | 15.03 | 1.81 |
|  | P:1 | 15.50 | 1.32 |
| Year×zone | D:1 | 88.54 | 30.92 |
|  | D:2 | 72.29 | 23.41 |
|  | D:3 | 90.29 | 32.51 |
|  | D:4 | 41.02 | 32.15 |
|  | D:5 | 10.66 | 30.42 |
|  | P:1 | 191.00 | 59.68 |
| Location×zone | D:1 | 47.81 | 23.76 |
|  | D:2 | 76.56 | 28.47 |
|  | D:3 | 7.99 | 11.45 |
|  | D:4 | 29.89 | 26.62 |
|  | D:5 | 218.89 | 117.61 |
|  | P:1 | 90.90 | 29.18 |
| Year×location×zone | D:1 | 104.20 | 14.29 |
|  | D:2 | 131.47 | 12.56 |
|  | D:3 | 140.74 | 20.25 |
|  | D:4 | 239.98 | 41.04 |
|  | D:5 | 189.22 | 46.19 |
|  | P:1 | 172.06 | 13.03 |
| Genotype×year×zone | D:1 | 5.53 | 0.85 |
|  | D:2 | 6.28 | 0.64 |
|  | D:3 | 5.82 | 0.93 |
|  | D:4 | 5.90 | 1.17 |
|  | D:5 | 1.81 | 0.99 |
|  | P:1 | 6.09 | 0.60 |
| Genotype×location×zone | D:1 | 1.59 | 0.71 |
|  | D:2 | 4.67 | 0.62 |
|  | D:3 | 1.80 | 0.98 |
|  | D:4 | 1.94 | 1.26 |
|  | D:5 | 3.65 | 1.33 |
|  | P:1 | 8.46 | 0.49 |
| Residual | D:1 | 25.02 | 0.99 |
|  | D:2 | 24.50 | 0.68 |
|  | D:3 | 29.75 | 1.33 |
|  | D:4 | 31.68 | 1.63 |
|  | D:5 | 24.40 | 1.64 |
|  | P:1 | 27.36 | 0.49 |

**Table S3b** Estimated correlations and their standard errors (in parentheses) of genotype×zone effects.

|  | D1 | D2 | D3 | D4 | D5 | P1 |
| --- | --- | --- | --- | --- | --- | --- |
| D1 |  |  |  |  |  |  |
| D2 | 0.9182  (0.0356) |  |  |  |  |  |
| D3 | 0.9505  (0.0349) | 0.9988  (NA) |  |  |  |  |
| D4 | 0.9515 (0.0357) | 0.9441 (0.0364) | 0.8501 (0.0483) |  |  |  |
| D5 | 0.9992  (NA) | 0.9706  (0.0361) | 0.8889  (0.0449) | 0.9987  (NA) |  |  |
| P1 | 0.7245  (0.1143) | 0.7831  (0.1124) | 0.9628  (0.0833) | 0.7113  (0.1261) | 0.715  (0.1211) |  |
